# Analysis of Tool Geometry for the Stamping Process of Large-Size Car Body Components Using a 3D Optical Measurement System

**DOI:** 10.3390/ma14247608

**Published:** 2021-12-10

**Authors:** Artur Rękas, Tomasz Kaczmarek, Michał Wieczorowski, Bartosz Gapiński, Michał Jakubowicz, Karol Grochalski, Dawid Kucharski, Lidia Marciniak-Podsadna

**Affiliations:** 1 Gestamp Polska Sp. z.o.o., Dzialkowcow Street 12, 62-300 Wrzesnia, Poland; arekas@pmc-smart.pl (A.R.); tomasz.kaczmarek@pl.gestamp.com (T.K.); 2 Division of Metrology and Measurement Systems, Institute of Mechanical Technology, Faculty of Mechanical Engineering, Poznan University of Technology, Piotrowo Street 3, 60-965 Poznan, Poland; michal.wieczorowski@put.poznan.pl (M.W.); karol.grochalski@put.poznan.pl (K.G.); dawid.kucharski@put.poznan.pl (D.K.); lidia.marciniak-podsadna@put.poznan.pl (L.M.-P.)

**Keywords:** sheet metal forming, stamping, 3D scanning, coordinate measuring techniques, quality control, at-line control

## Abstract

The paper presents a method for checking the geometry of stamped car body parts using a 3D optical measurement system. The analysis focuses on the first forming operation due to the deformation and material flow associated with stall thresholds. An essential element of the analysis is determining the actual gap occurring between the forming surfaces based on the die and punch geometry used in the first stamping operation. The geometry of car body elements at individual production stages was analyzed using an optical laser scanner. The control carried out in this way allowed one to correctly position the tools (punch and die), thus introducing the correction of technological parameters, having a fundamental influence on the specific features of the final product. This type of approach has not been used before to calibrate the technological line and setting of shaping tools. The influence of the manufactured product geometry in intermediate operations on the final geometry features was not investigated.

## 1. Introduction

**Figure 1:**
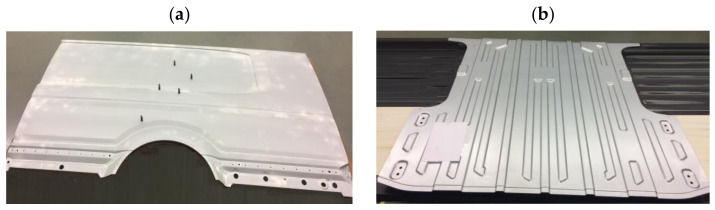
Large-size car body elements: (**a**) side of the vehicle, (**b**) floor.

The plastic forming of sheet metal in a multi-operation stamping process is essential in producing car body elements. Due to high-quality requirements, which the final products must meet, more and more emphasis is being placed on the dimensional and shape accuracy, allowing a correct final product [1,2,3,4]. The requirements concerning the accuracy of dimensions and shape are essential in the case of products such as cars [5,6,7], which are mass-produced, and their bodies consist of dozens of elements pressed from steel or aluminum sheets [8]. The correct quality and assembly of all the elements directly affect the safety during vehicle operation and the economy of its use [9]. Many of the external skin elements are large-sized, often exceeding the area of several square meters (Figure 1).

Obtaining spatial shapes of sheet metal products, due to the necessity of applying deep drawing together with cutting operations, is not possible only in one operation using one toolset [10]. Body parts are formed in a multi-operation stamping process, during which stamping and cutting operations are carried out, including the punching and cutting off technological allowances. From the point of view of geometric shaping, two technological operations are crucial: the first one is the deep drawing operation, during which the basic shape of the product is given, and the second one is the last drawing operation, during which the final shape of the product is given [11].

In production practice, there are many problems concerning the correct stamping of metal sheets. They may be based on tooling, gaps and tolerances, lubrication, working material properties and characteristics, etc. [10,11]. The most common procedure is the final product obtained after the whole stamping process is subjected to dimensional, shape, and visual inspection [12]. On its basis, individual operations of the forming process are corrected. This approach works well; however, it causes the necessity of a frequent correction, as the resultant inaccuracy of all operations must fit into the tolerance field adopted for the final product [13,14,15]. Precise matching toolset geometries at the production start-up stage are often time-consuming and require many attempts to correct errors. These corrections are often necessary, for example, after a change in the delivered batch of material or a change in thermal conditions in the production hall [16]. Control applied during the stamping process is also possible, but it requires tool design changes, which is expensive and time-consuming [17,18].

Quality requirements for manufactured car body products have necessitated a measurement method to analyze them in terms of shape and dimensions, often where a contact of the measuring tip is impossible or uneconomical due to measurement time. 3D scanning technology is increasingly used in the automotive industry [19,20] to control both the dimensions of objects with complex spatial geometry made of plastics [21,22,23] and body elements shaped in the stamping process [24]. Measurement systems equipped with optical scanners, many measurement points, and the lack of physical contact with the measuring element also provide the speed and repeatability of performed tests. Due to its advantages, 3D scanning technology is irreplaceable in many very different cases [25,26,27,28], and laser devices are much less sensitive to light reflections, which, in many cases, eliminates the need for troublesome surface dulling.

In this paper, the authors have proposed a new approach to dimensional and shape control after the first stamping operation and geometry measurement using an optical measurement system. The analysis of the geometry of car body parts stamped in the first crucial technological stage allows the adjusting of the stamping process so that geometrical features of the shaped product are in the middle of the accepted tolerance field. This leaves a much wider range for tool setting adjustments in subsequent operations, and in the case of thermal conditions change or material batch change, it causes, to a much lesser extent, the need for subsequent adjustments of their settings, significantly reducing downtime during the manufacturing process and reducing the number of parts not meeting accuracy requirements [29,30]. Such an approach is essential in the case of large-size components, as sheet-metal stamping requires tools with appropriate dimensions, which implies their high mass, translating into inertia during work movement. This causes problems with proper centric positioning of the punch and the die in relation to each other to ensure uniform material flow, providing proper shaping of the whole product in a given operation.

## 2. Methodology

**Figure 2:**
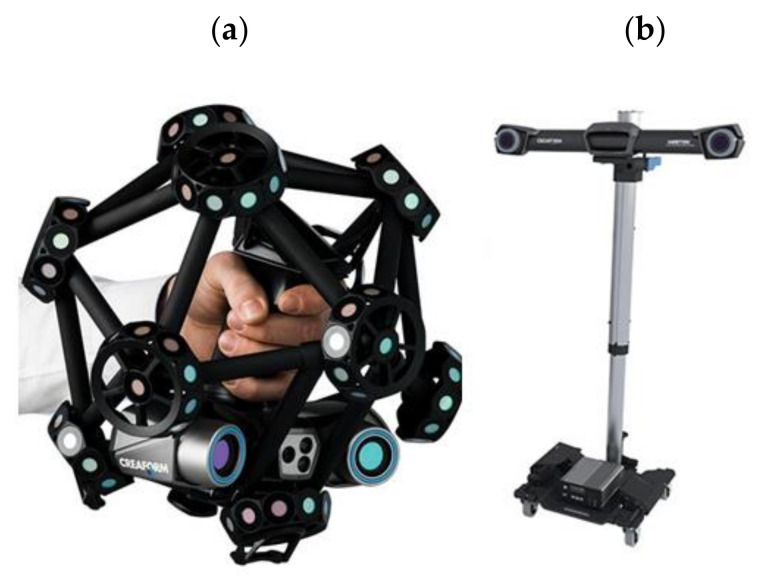
A scanner: (**a**) triangulation head, (**b**) C-track optical tracker [31].

The measurement of large components was carried out using a scanner with a triangulating laser head Creaform Metrascan 750 (Figure 2a) and an optical tracker Creaform C-Track next (Figure 2b) (Creaform - AMETEK Canada Limited Partnership, Levis, QC, Canada). The scanner head is characterized by a maximum measurement accuracy of up to 0.03 mm. The scanner allows the obtaining of a spatial image of the entire surface of the examined object with dimensions ranging from 0.2 m to 7 m. It is possible to obtain a color deviation map showing the actual distribution of product geometry deviations from the reference value or nominal model for the scans obtained.

**Figure 3:**
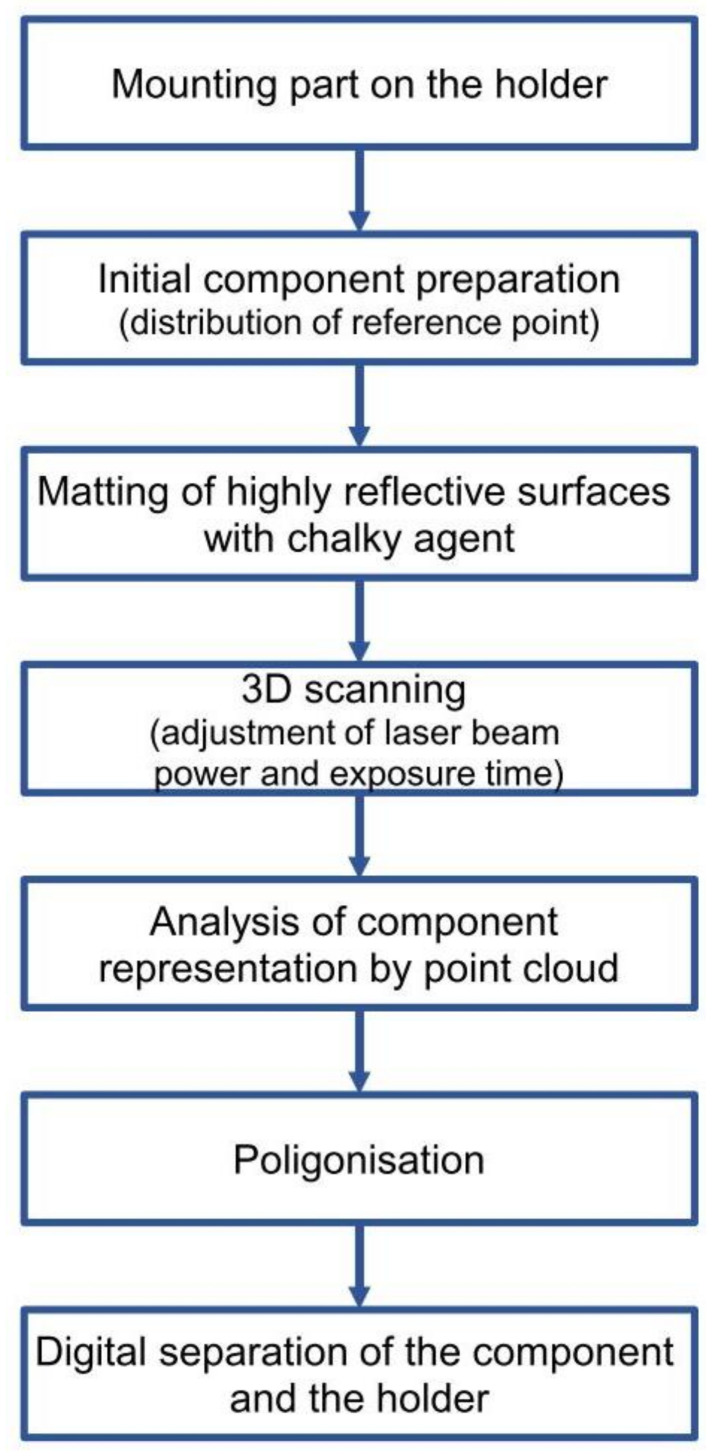
Sequences of measurement strategy.

The optical tracker was directed toward the test object to identify reference points on the test object and the laser scanner head during the tests. The test object was placed on specially prepared holders, ensuring its proper support. Measurements of the geometry of bulky products are difficult due to their low stiffness. Bulky products with low stiffness are subject to deformation, including material deflection under their weight. This is particularly evident in the case of the outer part of the car bonnet, where the central part of the product deflects, leading to a deformation of the entire product outline. In order to ensure stability and repeatability of the geometry measurements, dedicated measuring stations (gauges) were used. The measuring stations consisted of grips and elements supporting the product. Before the measurement, the product was placed in the grips and positioned in the characteristic places of the product to ensure geometric stability. In the case of the external element of the car bonnet, the product support elements were placed in the middle part of the measurement stand. Sequences of measurement strategy are shown in Figure 3.

In many cases, the support elements are adjustable, which allows for adjustment and setting in the nominal position according to the spatial CAD documentation of the product. This also enables their replacement in the case of wear or damage, thus avoiding expensive repair or replacement of the entire complex support system. Before the measurement, the test object was covered in difficult-to-measure areas with a chalky agent to reduce light reflections. On such a prepared surface, the optical system was calibrated in terms of, among others, the power of the laser beam illuminating the tested item and the exposure time. Data acquisition was carried out in VXelements software (Creaform - AMETEK Canada Limited Partnership, Levis, QC, Canada).

Large-size sheet metal parts, which exceeded the dimensions of the input sheet of 2 m^2^, were tested. Measurements of such components using optical scanners imply technical and methodological problems. One of the most significant challenges is the proper selection of the element’s mount, ensuring its stability during measurement data acquisition. The problem with the selection and implementation of such a system increases as the size of the element increases. Due to the size of the object, time is needed to scan the entire surface. In addition, pressed sheet metal components are, in many cases, flimsy, and deformations can occur due to both their own weight and shape. This is particularly noticeable with shallow stamped parts and flat surfaces where the product’s geometry does not provide adequate rigidity. Therefore, the fixturing system must provide support and clamping to stiffen the test object without distorting the results. The additional problem is that the mounting system obscures the areas to be measured. In these areas, measurement with an optical scanner is challenging, requiring additional processing of the point cloud and often leaving unmeasured fragments. In the case of optical measurements, it is also necessary to ensure the correct joining of individually measured fragments into one point cloud. Reference points must be used for mutual orientation of the tracker, scanner head, and measured object. In the case of large-size components, the tracker often does not cover the entire part to be measured. It must therefore be relocated. For this reason, reference points must be positioned in such a way that they can be continuously observed by the optical system of the tracker, especially if the tracker changes position. For these reasons, when scanning large components, the measurement strategy is crucial and, together with the correct preparation of the measurement space and the orientation of the measured component, ensures metrologically correct results.

## 3. Measurements and Analysis of Tool Geometry

**Figure 4:**
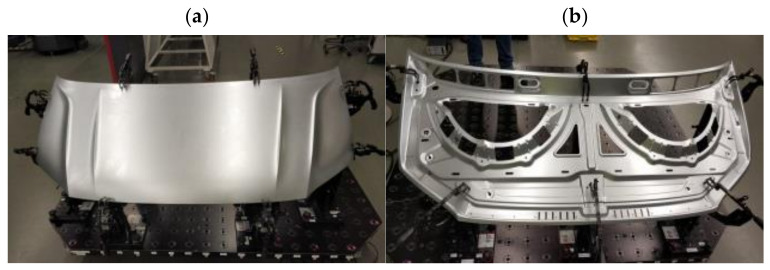
Tested products of the car bonnet after the last pressing operation element: (**a**) external, (**b**) internal.

Geometry measurements were carried out for two large-size car body elements manufactured in the stamping process from a 0.9 mm-thick steel sheet. One of the parts analyzed is the outer element of the car bonnet (Figure 4a), and the other is the corresponding inner reinforcement (Figure 4b).

In both cases, the production process includes six forming operations. The first operation is stamping, where the highest deformation is obtained in relation to the other forming operations. The next five operations are stamping processes combined with cutting operations.

In the initial stage of the stamping process, the sheet before introduction to the first forming operation was subjected to a washing process, and then a lubricating film was applied on its surface. Thus, the prepared sheet for both the external car bonnet and the internal car bonnet reinforcement was formed in the first operation with a pressing force of 4160 kN at a speed of 300 mm/s. In the remaining five operations, stamping and cutting was carried out, including punching and cutting off technological surpluses. The forming force ranged from 320 kN to 590 kN, and each operation was performed at a speed of 300 mm/s. The stamping process on the line was carried out at ambient temperature.

**Figure 5:**
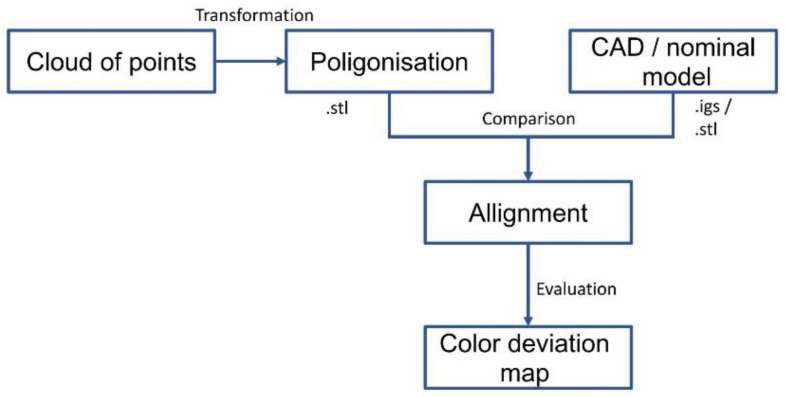
Data evaluation.

In the first stage of the study, the geometry of the finished part’s external and internal mask reinforcement was measured using an optical measurement system. The measured data were fitted to nominal data using the best fit method (Figure 5).

**Figure 6:**
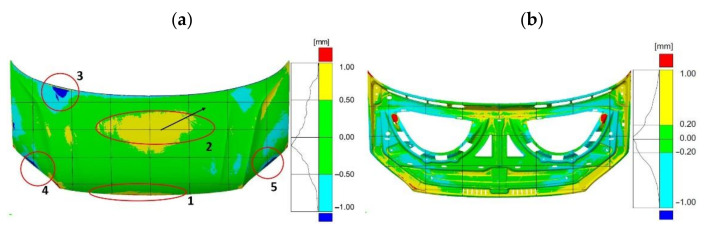
Distribution map of deviations in car bonnet dimensions made after the last pressing operation with respect to CAD model—element: (**a**) external, (**b**) internal.

The geometry measurement results allowed for the development of a 3D computer representation of the actual products. In order to evaluate the geometry of the finished parts, three-dimensional representations of scanned products after the final stamping operation were compared with spatial CAD models (Figure 6).

**Figure 7:**
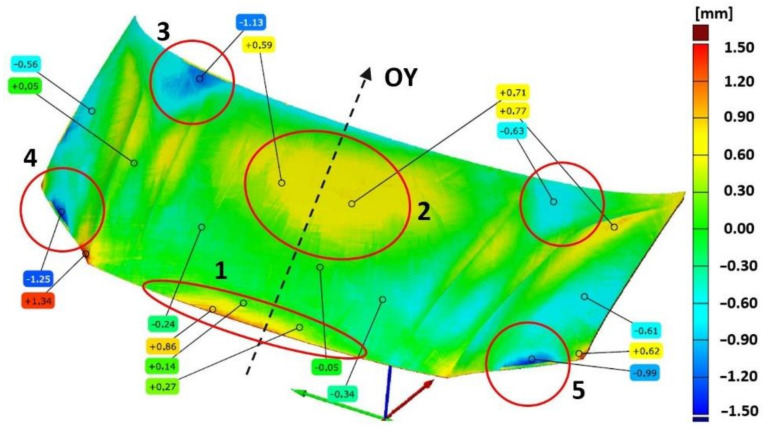
Distribution map of deviations in dimensions of the external car bonnet made after the last stamping operation with respect to the CAD model.

**Figure 8:**
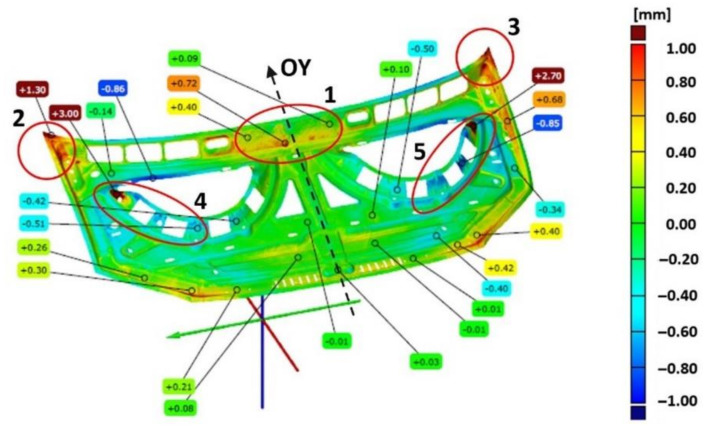
Distribution map of deviations in the inner dimensions of the car bonnet made after the last stamping operation with respect to the CAD model.

The required tolerances of the finished bonnet outer element (Figure 7) and bonnet inner reinforcement (Figure 8) are not the same in all marked areas. The center area of the outer mask element (2) has a defined dimensional tolerance of ±0.8 mm, while the other areas (1, 3–5) have tolerances of ±0.5 mm (Figure 7). Similarly, tolerances are adopted for the internal mask reinforcement in the middle area (1), where the tolerance is ±0.8 mm; in the other areas (2–3), the tolerance is ±0.5 mm (Figure 8).

The results of comparing the outer mask surface model geometry with the CAD model are presented in the form of a deviation map (Figure 7). The values of deviations in dimensions in characteristic areas of the external mask have been marked (Figure 7). The obtained measurements indicate the occurrence of significant differences in the deviation from the nominal value. The outer part of the mask is characterized by deviations ranging from −0.63 mm to +0.77 mm (Figure 6a). Excluding the area close to the front edge of the product, marked in Figure 4a with the number (1), the deviations are between −1.25 mm and +1.34 mm. In the middle area (2) of the product, the values of deviations are positive and range from +0.71 mm to +0.77 mm; however, their distribution is not symmetrical and there is a shift according to the direction marked by the arrow (Figure 6a). Much more significant asymmetry is observed in area (3) of the product part comparing the areas marked in Figure 7. The values of deviations in the marked areas (3) are negative and vary from −1.13 mm to −0.63 mm. The area (4) of the product (Figure 7) is characterized by larger negative deviations over a much larger area compared to the marked area (5) (Figure 6a and Figure 7). Other areas where symmetry was not maintained, mainly along the embossing edges, have also been marked.

In the case of the inner part of the mask, the comparison of the geometry of the scanned product with the CAD model is presented in Figure 8 in the form of a map of deviations with marked values. Measurements of the geometry of the inner part of the mask after the last stamping operation excluding the marked areas show deviations ranging from −0.4 mm to +0.4 mm (Figure 8). The area in the upper part of the product (1) near the axis of symmetry, in which the symmetry of the deviations’ distribution was not preserved, was determined. In the remaining areas, the values of deviations are symmetrically distributed with respect to the central axis (OY) of the element corresponding to the axis of symmetry of the vehicle. However, in the marked areas (2–5), significant deviations ranging from −0.86 mm to +3.00 mm are presented. The comparative analysis of the features of the geometry of the scanned finished products of the external surface and the inner part of the bonnet with the CAD model has shown the necessity to correct the geometry or the setting of the shaping tools in order to ensure the correct reproduction of the product geometry in accordance with the assumed dimensional tolerance and the CAD model.

**Figure 9:**
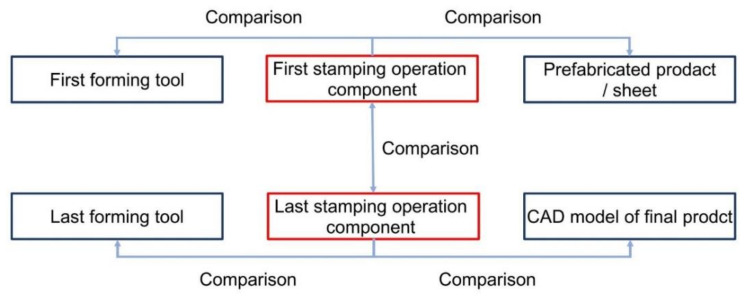
Methodology of analysis of forming tool geometry reproduction on final product.

In the next research stage, the geometry of the toolset used in the first stamping operation was analyzed. The forming of sheet metal products implies that the largest plastic deformations are obtained in the first stamping operation, which finally defined the research methodology, as shown in Figure 9.

**Figure 10:**
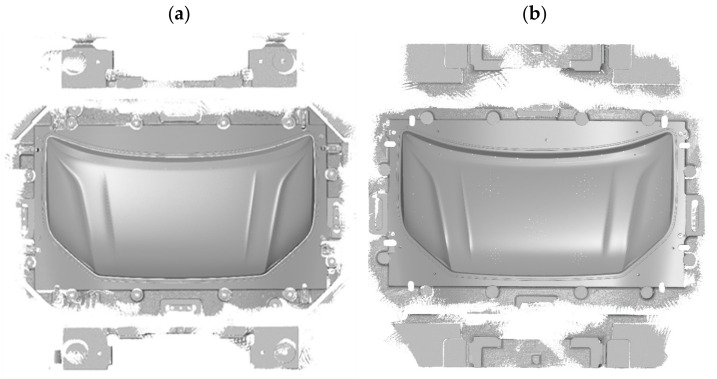
Tool geometry for stamping the inner mask component: (**a**) die, (**b**) punch.

A semi-finished product is produced with a designed geometry corresponding to the shapes of the punch and die geometries. The correct geometry and tooling adaptation allows the product to achieve the desired shape during forming. Therefore, discrepancies in the geometry, set-up, and fit between the die, punch, and press will result in increased dimensional deviations and, in extreme cases, excessive rubbing of the material, leading to cracking. The most common method for check tools is inking, in which blue ink is used to cover the blank and carry out the stamping process. The analysis of the quality of the tool coloring (inking) is the basis for correcting the tool geometry. A newer method is the method of tool geometry correction based on the analysis of scanning results. Despite advanced CAx techniques, the industrial practice has technical limitations that prevent the production of the die, punch, and ram from having a perfect geometry mapping to ensure proper material shaping. For this reason, the toolsets for each stamping operation are adjusted during the first production run. In order to identify the matching geometry of the die and punch, the die and punch surfaces were measured using a 3D optical measurement system (Figure 10).

**Figure 11:**
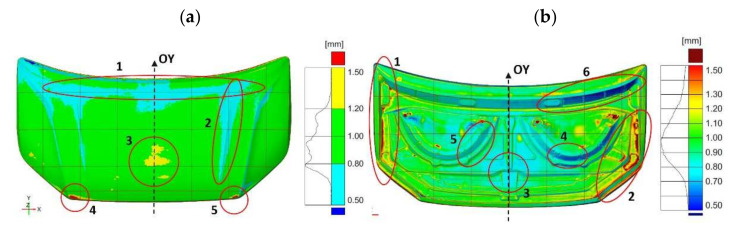
Distribution map of the gap between the die surface and the punch for the element: (**a**) external, (**b**) internal.

By comparing the results of the die and punch geometry measurements, the gap occurring between the forming surfaces was analyzed (Figure 11). The measurement of the gap between the die and the punch surface for the outer mask component (Figure 11a) indicates that in the upper part of the product (1) and along the embossing edges (2), the gap is smaller than the material thickness and ranges from 0.5 mm to 0.8 mm, and its distribution is not symmetrical with respect to the product axis. On the other hand, in the central part of the outer mask (3) and in the lower corners (4 and 5), there are areas where the distance between the die and the punch is larger up to about 50% of the material thickness. Heterogeneity in the distribution of the gap between the die and punch surface is also present in the inner component of the mask (Figure 11b). The outer areas (1 and 2) have a larger gap between the die and punch than the material thickness. On the other hand, in the middle area (3) and at the point of greatest deformation (4–6), the distance between the die and punch is smaller than the thickness of the feedstock. Moreover, the distribution of the distance between the die and the punch is not symmetrical. The map of the distance distribution between the die and the punch (Figure 11b) shows that the gap on the right side (4 and 6) is smaller, which may indicate a problem with the parallel positioning of the punch with respect to the die.

**Figure 12:**
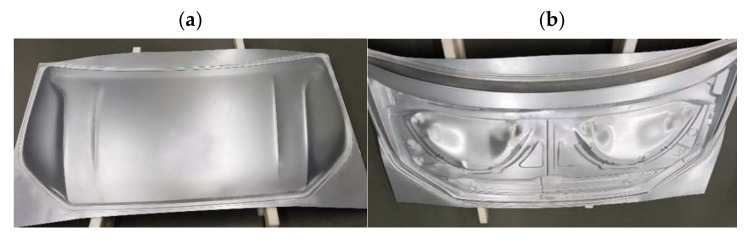
Tested car bonnet products after the first stamping operation element: (**a**) external, (**b**) internal.

**Figure 13:**
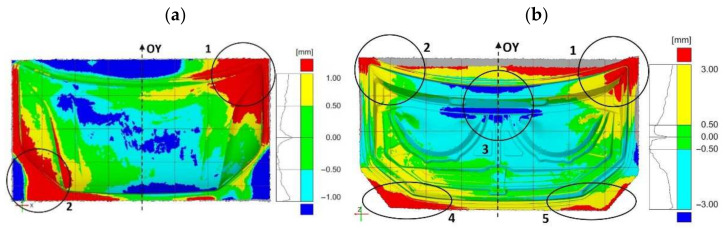
Map of the distribution of deviations between the geometry of the blank obtained in the first pressing operation and the die for a car bonnet element: (**a**) external, (**b**) internal.

In the next research stage, the geometry of semi-finished products after the first stamping operation (Figure 12) was compared with the die geometry. The determined deviations are a combination of manufacturing inaccuracies and changes occurring in the elastic deformation area. Taking into account all the components affecting the value of deviations, the analysis mainly focused on the symmetry of their distribution. In the case of the inner component of the bonnet, higher symmetry in the distribution of deviations can be observed than for the outer part of the bonnet (Figure 13).

The outer element of the mask has a smaller range of deviations than the inner element of the mask. However, in the case of the outer mask element, the asymmetric distribution of deviations is dominant (Figure 13a). In area (1) of the outer mask element, positive deviations dominate (Figure 13a). Similarly, area (2) is dominated by positive deviations occurring both in the area of the material that is cut-off as a technological surplus and in the blank subjected to further shaping operations. Positive deviations in the area of pull-through thresholds influence their lower effectiveness, as they hold, to a lesser extent, the material that should be shaped by stretching. The inner component of the mask (Figure 13b) has larger positive deviations in area (1) compared to the symmetrical area (2). In the middle area (3), there are negative deviations, but their distribution maintains symmetry. Comparative analysis of the blank geometry after the first pressing operation with the die geometry for the inner mask element shows larger deviations in area (4) than the symmetrical area (5).

**Figure 14:**
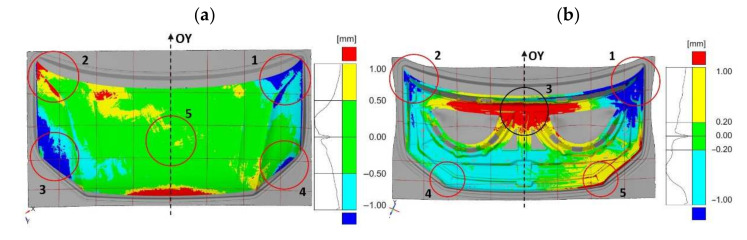
Map of deviations between the product obtained after the last pressing operation and the component after the first pressing operation for the component: (**a**) external, (**b**) internal.

In order to determine the direct influence of the element geometry after the first forming operation on the final product, a comparative analysis of the element geometry obtained after the first forming operation with the product after the last operation was carried out (Figure 14). In the case of the outer element, the occurring deviations and their asymmetry of distribution (Figure 14a) are a consequence of the distribution of deviations when comparing the geometry of the blank after the first stamping operation with the geometry of the die (Figure 13a). The mask outer element is characterized by an asymmetric distribution of deviations in the outer areas (Figure 14a). In the upper area (1) of the mask outer element, negative deviations of less than −1.0 mm are present, while in the symmetrical area (2), positive deviations exceed 1.0 mm (Figure 14a). In area (3), there are much larger negative deviations compared to the symmetrical area on the right (4) (Figure 14a). The central area (5) of the part is dominated by areas with a deviation in the range of ±0.5 mm (Figure 14a). In addition, in the case of the mask inner element, the consequence of the distribution of deviations and their asymmetry (Figure 14b) is the occurrence of asymmetry in the distribution of deviations when comparing the geometry of the blank after the first pressing operation with the geometry of the die (Figure 13b). Measurements of the inner element of the mask showed that area (1) has smaller negative deviations, less than −1.0 mm compared to the symmetrical area (2) (Figure 11b). In the central area (3), there are positive deviations exceeding +1.0 mm. The asymmetry of the distribution of the deviations was determined in the lower area (4), where the deviations are negative in the range from −1.0 mm to −0.2 mm, and the deviations in area (5) are positive and range from +0.2 mm to +1.0 mm (Figure 14b).

**Figure 15:**
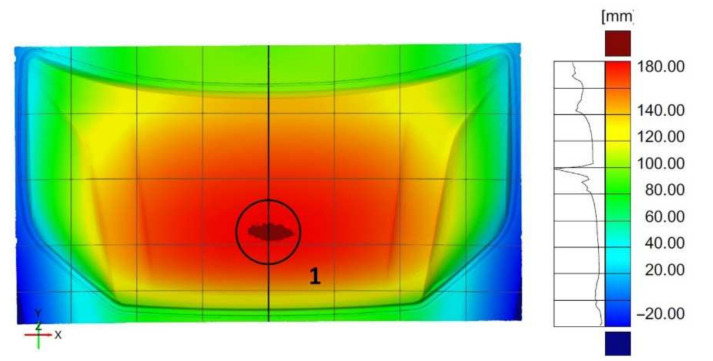
Map of displacement distribution between the flat sheet and the molded part resulting from the first stamping operation for the external element of the mask.

Measurements of the geometry of the final product and the die, punch, presser, and blank after the first pressing operation with the use of a 3D optical measurement system and their appropriate combination enabled the determination of the geometric deviations. The analysis of the deviations and their distribution with respect to the axis of symmetry allowed for correcting the toolset used in the first pressing operation, as well as for introducing corrections in the geometry of the tools themselves. In order to verify the corrections made to the geometry of the tools used in the first pressing operation, the displacement of the flat sheet from which the product is pressed was measured. In the case of the outer mask element, the highest displacement of 185 mm was determined in the central area (1) of the product (Figure 15).

The analysis of the displacement distribution showed symmetry with respect to the product axis due to the accurate mapping of the tool geometry. The edges of the feed material in the areas outside the string threshold were not deformed, indicating the effectiveness and uniformity of the string thresholds. The thrust thresholds were used to prevent the material from flowing, and the deformations obtained result from the material stretching (Figure 15).

**Figure 16:**
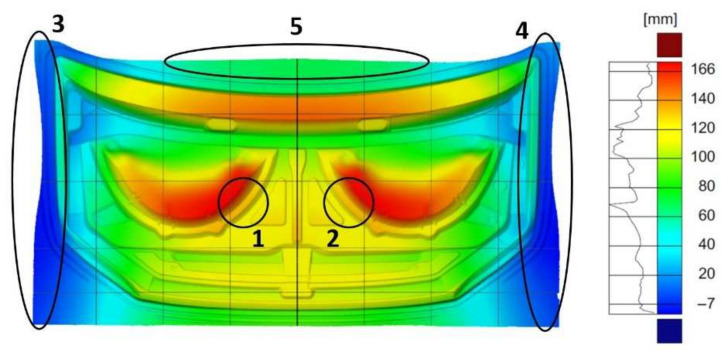
Map of displacement distribution between the flat sheet and the molded part resulting from the first stamping operation for the inner element of the mask.

The effectiveness of the corrections made to the tool geometry in the first stamping operation was analyzed for the inner mask component, and the displacement of the flat sheet from which the product is stamped was measured. The largest displacements relative to the input sheet were measured in areas (1 and 2), amounting to 166 mm with symmetrical distribution (Figure 16). The displacement measurement results obtained showed that the tool geometry was accurately reproduced and that the symmetry with respect to the product axis was maintained. The edges of the feed material in the areas beyond the draft threshold (3–5) were symmetrically deformed. The visible deformation effect (3–5) was due to the controlled flow of the material in the draft threshold area (Figure 16).

## 4. Discussion

Using an optical measurement system to measure geometries and their comparison to determine deviations supports the process of the precise geometry matching of toolsets at the production start-up stage. Geometry discrepancies were identified by comparing geometry models of scanned products after the final stamping operation with spatial CAD models. The determined deviations indicated areas that required correction for correct shape matching. For the outer part of the mask, areas (1, 3–5) were indicated (Figure 7) that required correction due to exceeding the required dimensional tolerance of ±0.5 mm. The inner part of the mask in places outside the marked areas shows deviations in the range of ±0.4 mm that meets the requirements of tolerance ±0.5 mm (Figure 8). In addition, area (1) with positive deviations of up to +0.77 mm met the ±0.8 mm tolerance requirements for this area. Areas (2–5) were determined for the inner mask component that required correction due to exceeding the required dimensional tolerance of ±0.5 mm (Figure 8).

Comparative studies of scanned surfaces from selected stages of the stamping process allowed one to indicate the place and range of introduced corrections on the basis of correlation analysis of the determined deviation distributions. The results of the distribution of deviations of the product dimensions after the last pressing operation against the CAD model (Figure 7) were correlated with the map of the gap distribution between the die surface and the punch (Figure 11a) in the determined areas. The results of the correlation allowed the determination of the convergent areas and the extent of divergence and indication of nonuniformity in tool alignment. Then, the obtained results were correlated with the distribution of the deviation of the blank and the die for the first stamping operation (Figure 13a). The areas and ranges of divergences were determined, and the influence of technological allowances and draft thresholds on the distribution of deviations was analyzed. The correlation results allowed the specification of the influence of settings and parameters of the first forming operation on the range of deviations in the analyzed areas, and to assess the effectiveness of draft thresholds and technological allowances. The correlation of the results obtained on the basis of the above analysis with the distribution of deviations between the product and semi-finished product (Figure 14a) served to verify the adopted assumptions and to determine the place and scope of corrections in the press settings and tool geometry.

The test results for the external part of the car bonnet allowed the development of the above procedure for determining the distribution of deviations, determining the areas outside the tolerance range, quantitative evaluation of deviations, and correlation of the obtained results at individual stages of testing. In order to validate the measurements and the method, tests were performed for the internal reinforcement of the car bonnet according to the adopted procedure. The results obtained allowed us to indicate the place and range of corrections introduced in the settings of the press and the geometry of tools.

## 5. Conclusions

The research was realized in order to support error correction in the production process. Production start-up requires adjustment of toolset geometry and press settings in the production line. In addition, in the case of products already implemented into production, errors occur when the delivered batch of material is changed and when the process conditions are changed. In addition, errors need to be corrected when the production line is changed.

When the comparative analysis of the product geometry with its CAD model shows that the product does not meet the tolerance requirements, it is necessary to intervene in the production process. In production practice, the correction of errors is performed by repeated trials with minor changes. The analysis of the observed effects of the changes is the basis for introducing new changes and making another test until the required quality of the product is achieved. The cyclic introduction of changes is a time-consuming and thus costly process. The above method of correcting errors does not ensure that it is an optimal solution.

In the performed research, necessary corrections to the molding process were introduced in two stages. In the first stage of the research, the press settings were corrected. The press settings were corrected based on the deviations’ distribution analysis and regarding the preservation of symmetry in the analyzed distributions. It was essential to operate on numerical values, which indicated the place and the value of the press setting correction. The second stage of error correction was corrections of tool geometry. Polishing the surface in the indicated places based on the analysis of the distribution of deviations, mainly in the gap occurring between the shaping surfaces in the first pressing operation, allowed one to obtain the expected effect.

The developed method significantly accelerates the error correction process and specifies the areas and scope of correction. Investigations of tool and blank geometry after the first stamping operation using the optical measurement system allowed one to indicate the scope of press setting changes and toolset adjustments. The analysis of the deviations’ distribution allowed the determination of the areas of the high variability of dimensional deviations and significant asymmetry of distribution requiring correction.

## Data Availability

Data available on request due to privacy restrictions.
